# Hemothorax With White-Out Lung After Intrapleural Tissue Plasminogen Activator and Deoxyribonuclease Therapy in a Patient With Complicated Parapneumonic Effusion

**DOI:** 10.7759/cureus.26208

**Published:** 2022-06-22

**Authors:** Arjun Mainali, Samaj Adhikari, Tutul Chowdhury, Nicole Gousy, Roshan Bisural, Saujan Devkota, Ambika Devi Kaphle Bastola

**Affiliations:** 1 Internal Medicine, Interfaith Medical Center, Brooklyn, USA; 2 Medicine, American University of Antigua, New York, USA; 3 Internal Medicine, Nobel Medical College Teaching Hospital, Biratnagar, NPL

**Keywords:** complicated parapneumonic effusion, dnase, tissue plasminogen activator (tpa), white-out lung, hemothorax

## Abstract

Tissue plasminogen activator (tPA) and recombinant deoxyribonuclease (DNase ) are used in treating pleural infection due to their mucolytic activity by effectively reducing pleural fluid viscosity. The combination of tPA and DNase has attracted considerable interest as an alternative to surgical intervention for treating complicated parapneumonic effusion in high-risk patients who are not good candidates for surgery. However, intrapleural hemorrhage has been reported as a villainous outcome in a few cases which needs to be considered as a differential diagnosis with sudden clinical deterioration after the therapy. Here, we report the case of a patient who presented with pneumonia and later developed a large right complicated parapneumonic pleural effusion. A chest tube was placed with drainage of fluid while tPA and DNase were also considered as an additional treatment module. Following the first dose of DNase and tPA, the patient developed hypoxemia with hypotension and was found to have rapid development of white-out right hemothorax.

## Introduction

Intrapleural tissue plasminogen activator (tPA) and deoxyribonuclease (DNase) administration is a unique modality for treating complicated pleural effusion. Administration of fibrinolytic agents can help defer surgery in select patients. Despite their wide usage, these fibrinolytic agents rarely cause intrapleural hemorrhage leading to hemodynamic instability in patients [1,2]. Local bleeding and chest pain are commonly reported adverse effects [3]. Intrapleural administration of tPA in obstructed indwelling catheters leading to improvement in catheter drainage was previously reported [4,5]. Here, we present a case of white-out lung due to intrapleural hemorrhage after the administration of tPA and DNAse in a patient previously not on any anticoagulation, with complicated parapneumonic effusion.

## Case presentation

A 57-year-old female with a medical history of hypertension, chronic kidney disease stage 3A, opioid use disorder on methadone maintenance program (MMTP), heart failure with reduced ejection fraction, and hypothyroidism presented to the emergency department (ED) with complaints of generalized diffuse headache for three days after she ran out of her hypertensive medication. She reported a dry cough, shortness of breath on exertion, and bilateral swelling of the legs for four to five days. She denied chest pain, palpitation, orthopnea, paroxysmal nocturnal dyspnea, and fever. Reviews of systems were otherwise negative. She had a smoking history of 30-pack-years, denied alcohol use, reported to be on MMTP, and had been taking methadone 60 mg daily. All other histories were unremarkable.

On the physical examination, she was in mild distress due to a diffuse headache. Triage vitals showed blood pressure of 205/140 mmHg, a pulse of 70 beats/minute, a temperature of 36.8°C (98.2°F), respiratory rate of 17 breaths/minute, and saturating oxygen at 99% at room air. The chest examination showed bilateral vesicular breath sounds with mild basal crepitation. The cardiovascular examination showed a normal S1/S2, regular rate and rhythm with no murmur, rubs, or gallops. She had bilateral pitting edema. All other physical examinations were unremarkable. Appropriate lab work was performed (Table 1).

**Table 1:** Initial lab work findings during patient admission.

Test	Reference range and units	Values
White blood cell count	4.5–11.0 × 10^3^/µL	10.8
Hemoglobin	11.0–15.0 g/dL	9.7
Hematocrit	35–46%	30.5
Mean corpuscular volume	80–100 fL	75.9
Reticulocyte percent	0.5–2%	1.68
Lactate dehydrogenase	125–220 U/L	335
Folate	2.76–20.00 ng/mL	6.49
Vitamin B12	239–931 pg/mL	766
Ferritin	11.20–264.00	88.80
Total iron-binding capacity	240.00–450.00	441.59
Iron saturation %	%	20
Transferrin	206.00–381.00	315.42
Platelets	130–400 × 10^3^/µL	268
Blood urea nitrogen	7.0–18.7 mg/dL	36.6
Creatinine	0.57–1.11 mg/dL	1.21
Estimated glomerular filtration rate	≥90.0 mL/minute/1.73m^2^	52.3
Sodium	136–145 mmol/L	136
Potassium	3.5–5.1 mmol/L	4.4
Carbon dioxide	22–29 mmol/L	25
Total bilirubin	0.2–1.2 mg/dL	1.1
Alanine aminotransferase	10–55 U/L	<10
Aspartate aminotransferase	5–34 U/L	21
Alkaline phosphatase	40–150 U/L	91
Albumin	3.5–5.2 g/dL	3.9
Phosphorous	2.3–4.7 mg/dL	3.8
Intact parathyroid hormone	7.5–53.5 pg/ml	18.0
Calcium	8.4–10.2 mg/dL	8.9
Brain natriuretic peptide	10–100 pg/mL	3574
Lactate	0.50–1.90 mmol/L	3.79
High-sensitivity troponin	0–17 ng/mL	52.7
Prothrombin time	9.8–13.4 seconds	13.4
International normalized ratio	0.85–1.15	1.18
Partial thromboplastin time	24.9–35.9 seconds	28.7
Vitamin D 25-hydroxy	30–100 ng/mL	32

**Figure 1:**
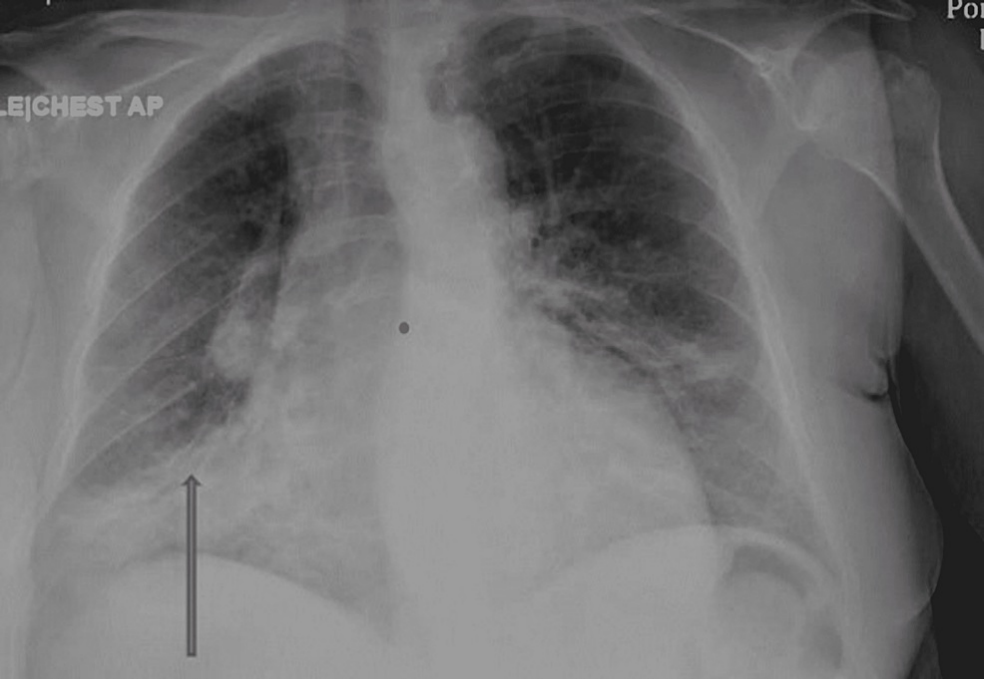
Portable chest X-ray anteroposterior view showing perihilar and bilateral lower infiltrates more on the right zone (arrow).

Urine toxicology was positive for benzodiazepine, methadone, and opiates. Urine analysis was within the normal limit. An electrocardiogram (EKG) normal sinus rhythm, heart rate of 92, QTc 460, without significant ST/T wave changes. Chest X-ray showed bilateral infiltrates more on the right lower zone (Figure 1).

The patient received one dose of labetalol 10 mg and amlodipine 10 mg and was started on Lasix 40 mg twice daily, aspirin 81 mg daily, and Lipitor 40 mg daily. After two days, Lasix was discontinued as kidney function started deteriorating. Blood pressure was optimized with nifedipine and hydralazine. Due to symptoms of mild cough and chest X-ray findings suggestive of community-acquired pneumonia, she was started on ceftriaxone 1 g daily and doxycycline 100 mg twice daily. After serum mycoplasma, immunoglobulin (Ig)M, and urine *Legionella *antigen were negative. Hence, doxycycline was discontinued. Troponin trended down, and repeated EKG was not significant for any ischemic changes. An echocardiogram showed an ejection fraction of 41%. She underwent nuclear medicine myocardial perfusion spect stress and rest test which revealed a small inferior wall fixed defect with no stress-induced reversible ischemia. Cardiac catheterization was done which showed diffuse triple vessel disease not amenable to percutaneous coronary intervention.

**Figure 2:**
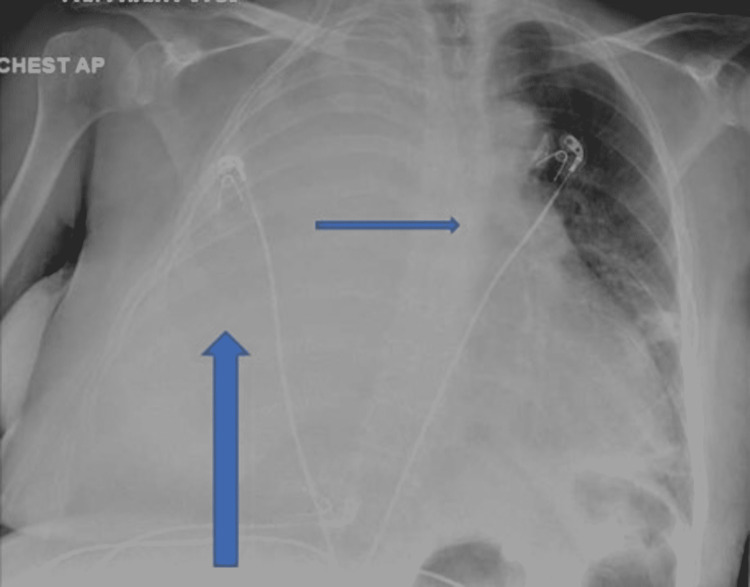
Portable chest X-ray anteroposterior view showing almost complete opacification of the right hemithorax with a mediastinal shift to the left (blue arrows).

After three days of admission, the patient started desaturating and was started on the nasal cannula and changed to a non-rebreather and later to bilevel positive airway pressure (BIPAP) after she failed to improve. White blood cell count trended up to 22.9 with procalcitonin of 0.64, and arterial blood gas (ABG) analysis was consistent with hypoxemia. A repeat chest X-ray showed almost complete opacification of the right hemithorax with a mediastinal shift to the left, suggestive of large pleural effusion (Figure 2). Antibiotics were changed to meropenem and vancomycin. Two sets of blood cultures were done which showed no growth. The patient later went into respiratory distress and was intubated and put on a mechanical ventilator.

Diagnostic thoracentesis with the placement of a chest tube was done for continuous drainage of pleural effusion. Pleural fluid analysis showed exudative effusion with features of complicated parapneumonic effusion. The pleural fluid analysis findings are shown in Table 2.

**Table 2:** Results of the pleural fluid analysis obtained during patient admission. WBC: white blood cell; RBC: red blood cell

Test (pleural fluid)	Reference range and units	Values
Appearance		Cloudy
pH	Not established	7.3
WBC	<200 WBC/µL with <25% neutrophil	9,600 with 85% neutrophil
RBC		4000
Protein	g/dL	5.6
Albumin	g/dL	1.7
Glucose	65–95 mg/dL	<2
Lactate dehydrogenase	IU/L	2,284
Gram stain		Moderate WBC. No organism
Culture		No growth
Cytology		Negative for malignant cells

**Figure 3:**
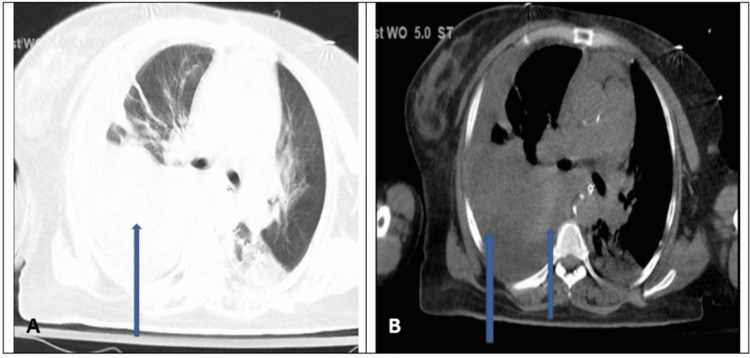
CT of the chest showing dense right lower lobe consolidation (blue arrow) (A) and large pleural effusion with a hyperintense lesion on the fluid (blue arrow) (B), suggestive of early septa formation. CT: computed tomography

Repeat serum total protein and lactate dehydrogenase (LDH) were 6.3 g/dL and 288 U/L, respectively. Pleural fluid protein/serum protein was >0.5 and pleural fluid LDH/serum LDH was >0.6. Based on the Light’s criteria and very high LDH of >1,000, pleural fluid was consistent with exudative pleural fluid with complicated parapneumonic effusion. Chest computed tomography (CT) was repeated which showed dense right lower lobe consolidation (Figure 3A) with a possible septum on the large pleural fluid (Figure 3B), suggestive of early septa formation with loculation.

**Figure 4:**
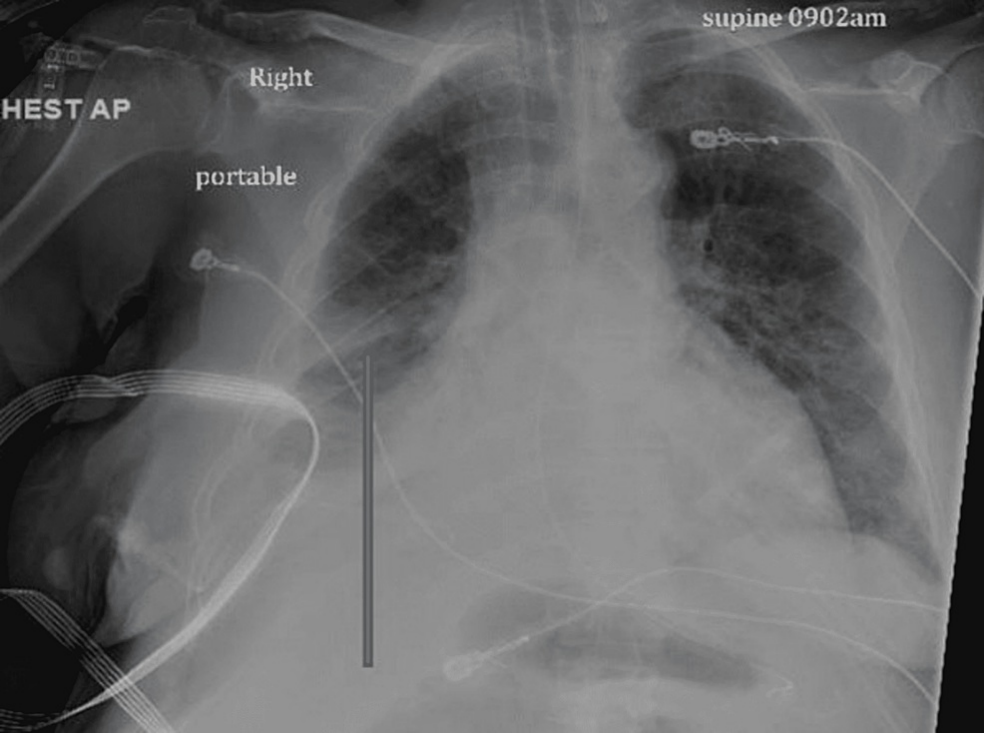
Portable Chest X-ray anteroposterior view taken after three days of chest tube drainage showing significant improvement compared to previous imaging (arrow).

Around 3.5 L of fluids were drained in two to three days. Chest X-ray repeated after three days showed improvement, as described in Figure 4.

**Figure 5:**
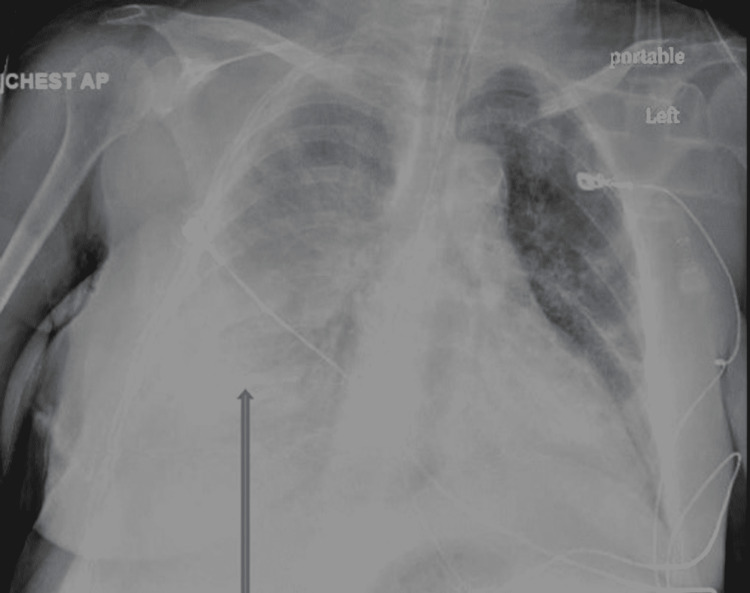
Portable Chest X-ray anteroposterior view showing a white-out right hemithorax suggestive of right hemothorax after DNase/tPA therapy (arrow). DNase: deoxyribonuclease; tPA: tissue plasminogen activator

**Figure 6:**
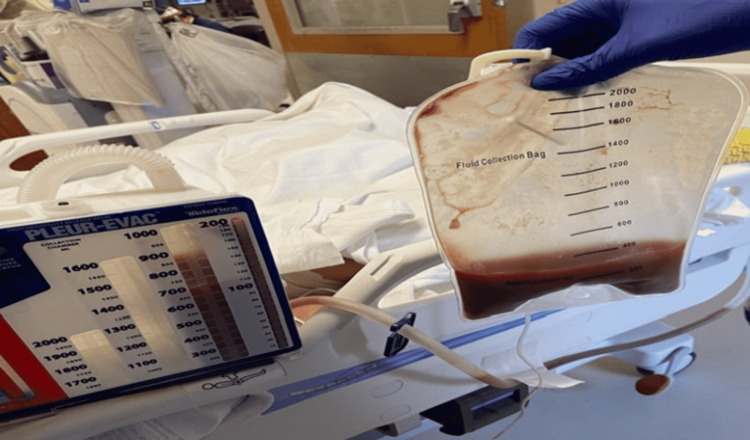
Bloody fluid collected from the chest tube after the patient became hemodynamically unstable suggestive of right hemothorax after tPA therapy. tPA: tissue plasminogen activator

Her renal functions also started deteriorating with a decrease in urine output and an increase in urea level of 88 mg/dL and creatinine of 5.86 mg/dL (estimated glomerular filtration rate: 9.6) with refractory hyperkalemia and potassium levels up to 7 mmol/L even after multiple medical cocktail therapy (calcium gluconate, dextrose, regular insulin, albuterol nebulization); hence, hemodialysis was started. Based on the pleural fluid analysis and repeat CT of the chest, she had a higher risk of developing loculated pleural effusion. Because surgical options would have been futile considering her comorbidities with diffuse triple vessel disease and chronic kidney disease on new initiation of hemodialysis, nonsurgical options with DNase and tPA were considered. DNAse 5 mg intrapleural followed by tPA 10 mg was given at one-hour intervals followed by clamping of a chest tube. After 30 minutes of tPA therapy, she started desaturating to 85-88%, with a drop in blood pressure of 88/52 mmHg. The fluid challenge with a 250 mL bolus was done, and she was started on norepinephrine after her blood pressure did not improve. Meanwhile, the chest tube was unclamped, and the chest X-ray was repeated, which showed a white-out right lung, as shown in Figure 5. The chest tube was draining bloody fluid, as shown in Figure 6, which was highly suggestive of right hemothorax after tPA therapy.

**Figure 7:**
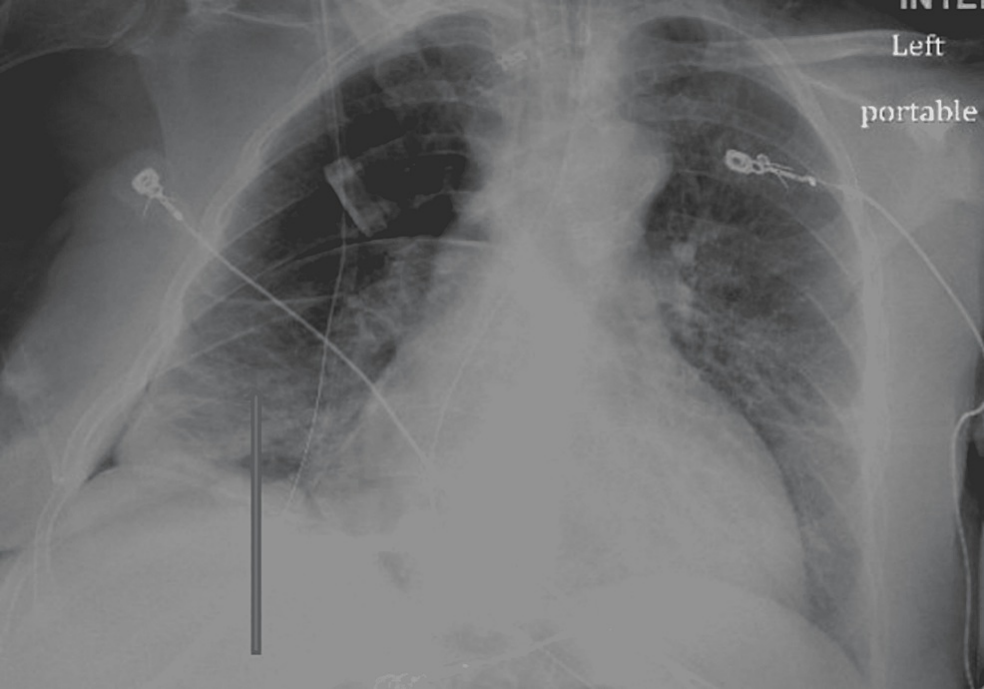
Portable chest X-ray anteroposterior view showing significant improvement in the right hemithorax (arrow).

Hemoglobin/Hematocrit was closely monitored, with a drop in hemoglobin to 7.8 g/dL after two days from 9.7 g/dL. She was transfused one unit of packed red blood cells to maintain hemoglobin above 8 g/dL in view of diffuse triple vessel coronary artery disease. She completed three days of treatment with tPA/DNase with no further episodes of bloody drainage. The patient clinically improved with resolution of leukocytosis and significant improvement in the chest X-ray (Figure 7). She was extubated after eight days. Antibiotics were continued for 21 days. Her renal function also improved after four sessions of hemodialysis, and creatinine returned to the baseline without further need for hemodialysis.

## Discussion

Complicated parapneumonic effusion and empyema represent a spectrum of infections within the pleural space [1]. In patients with complicated parapneumonic effusions or empyema, drainage is recommended as soon as possible to maintain infectious source control [1]. Drainage is especially stressed in patients with empyema which carries a higher risk and poorer patient outcomes. Specifically, loculated effusions, large free-flowing effusions, or effusions with a thickened pleural membrane are indications for drainage along with broad-spectrum antibiotics until a causative organism is identified [1,2]. Treatment failure of complicated parapneumonic effusion is defined as a failure to improve, or worsening effusion or leukocytosis is appreciated. This can indicate either insufficient organism coverage or inadequate effusion drainage. In either case, failure to improve or signs of worsening effusion are indications for surgical intervention via video-assisted thoracic surgery (VATS) [2,3]. However, an additional approach in this scenario would be to administer intrapleural tPA with adjuvant DNase. This strategy is particularly indicated when imaging reveals sufficient catheter placement within the pleural space where the administration of tPA/DNase would be effective. Due to the evasiveness of VATS, this treatment approach is preferred in patients who may not be good surgical candidates [2,3].

The second Multicenter Intrapleural Sepsis Trial (MIST-2) studied the effects of administering either intrapleural tPA or DNase separately or in combination to randomized patients with complicated parapneumonic effusion and evaluated their prognosis, length of hospitalization, and the need for VATS [4]. One of the major findings of this study was that, while the combination of medications reduced the need for VATS, the development of severe intrathoracic bleeding was seen only in patients who received a combination of medications. This study saw a rate of intrapleural bleeding of approximately 3.8% [4,5].

Since the study, follow-up reports have shown a variable range of hemothorax development or white-out lung in approximately 1.8-10% of those who received intrathoracic tPA with or without DNase [5]. Interestingly, tPA with concordant DNase administration on anticoagulant medication showed the development of hemothorax at an exponentially higher rate of 28-33% [5]. However, the risk of hemothorax was not shown to be increased in those who received aspirin, clopidogrel, or subcutaneous heparin [5,6]. While there is no clear mechanism of how specific anticoagulants increase the risk of hemothorax in these patients, it is clear that there is a synergistic link [7].

While the mechanism of action for how tPA and DNase work in increasing effusion drainage remains unclear, the major theory states that tPA breaks down loculations within the pleural space and DNase decreases effusion viscosity [2,8]. Additionally, the optimal dosages, frequency, and timing for tPA and DNase administration remain unclear. Currently, there is no clear link between the dosages of tPA and DNase and the development of intrapleural bleeding [2,4-7].

## Conclusions

The use of tPA or DNase as an intrapleural fibrinolytic therapy is an alternative to surgical treatment for complicated parapneumonic effusion. Physicians need to be vigilant in administering fibrinolytics as they can lead to severe hemodynamic instability. Further studies are warranted to determine the optimal dose and clinical criteria for the administration of fibrinolytics in patients with complicated parapneumonic effusion.
